# Erector spinae density as a predictor of early cardiac complications in patients with stroke

**DOI:** 10.3389/fcvm.2026.1878270

**Published:** 2026-08-12

**Authors:** Kun Ding, Yue Lin, Qing Zhu, Xiao Liu, Peidong Cao, Linlin Liang, Mingyue Cui, Jingwei Ni

**Affiliations:** 1 Taicang Loujiang New City Hospital (Ruijin Hospital, Taicang), Suzhou, China; 2 Zhejiang Provincial People’s Hospital, Hangzhou, China

**Keywords:** computed tomography, erector spinae muscle density, nutritional status, risk stratification, stroke-heart syndrome

## Abstract

**Rationale and objectives:**

The relationship between erector spinae muscle density (ESMD) and early cardiovascular complications in patients with stroke remains unexplored. Therefore, this study aimed to evaluate the predictive value of ESMD, assessed through chest computed tomography (CT), for the incidence of stroke–heart syndrome (SHS) in patients with stroke.

**Materials and methods:**

Clinical data of 422 patients with stroke from Zhejiang Provincial People's Hospital (January 2023–March 2024) were retrospectively analyzed. Patients were grouped based on the presence of SHS within 72 h of admission. Logistic regression analyses identified predictors, and a nomogram integrating ESMD was constructed. Model performance was evaluated through receiver operating characteristic (ROC) curves, calibration plots, and decision curve analysis.

**Results:**

Independent predictors of SHS included the ESMD, serum albumin level, estimated glomerular filtration rate, prothrombin time, and fibrinogen level (*P* < 0.001). Lower ESMD (<34.39 HU) significantly correlated with a higher SHS risk, with ROC analysis showing an area under the curve (AUC) of 0.741. The ESMD-integrated nomogram demonstrated excellent predictive performance (AUC = 0.815) and clinical utility.

**Conclusion:**

ESMD obtained from chest CT is a strong predictor of SHS, offering a practical, non-invasive marker for early risk stratification and management. The proposed nomogram provides an effective tool for identifying and managing patients at high risk of SHS.

## Introduction

Secondary cardiac injuries are frequently observed in patients with stroke and represent a significant clinical concern (1). Cardiovascular disease (CVD) is the second leading cause of post-stroke mortality (2). First reported by Byer et al. in 1947, cerebrovascular disease has been linked to myocardial injury and arrhythmias (3). Stroke can lead to various cardiac complications, including myocardial injury, arrhythmias, and cardiac insufficiency. The term “stroke–heart syndrome” (SHS) was introduced in 2018 to collectively describe these complications (4). While the underlying mechanisms of SHS remain under investigation, inflammation, immune dysregulation, and central autonomic network disturbances are increasingly recognized as key contributors (5, 6).

Nutritional status plays a pivotal role in overall health and disease progression (7). High nutritional risk, characterized by factors such as low serum albumin levels, unintentional weight loss, or reduced muscle mass, is associated with an increased incidence of stroke and can predict poor stroke outcomes (8, 9). However, traditional markers for evaluating nutritional status, such as body mass index (BMI) and waist circumference, are insufficient (10), especially in the older adult population, due to changes in body composition and increased fat infiltration. In addition, inflammation related to adiposity is not adequately captured by BMI or waist circumference alone (11, 12). A decline in nutritional status is often reflected by changes in muscle mass markers (13), which indicate both skeletal muscle quality and intramuscular fat volume. Low muscle density is a marker of increased fat infiltration (14, 15). Excess adipose tissue places the body in a state of chronic low-grade inflammation (16), which is believed to play a pivotal role in the pathogenesis of SHS.

In our preliminary observations, we found that patients with stroke with low erector spinae muscle density (ESMD) were more likely to develop secondary cardiac injuries. However, the predictive value of ESMD for SHS in post-stroke patients has not been systematically explored.

This study aimed to evaluate the predictive significance of ESMD for SHS in patients with stroke. Additionally, we developed a nomogram to assist clinicians in predicting the incidence of SHS and optimizing patient management strategies.

## Material and methods

### Study population

**Figure 1:**
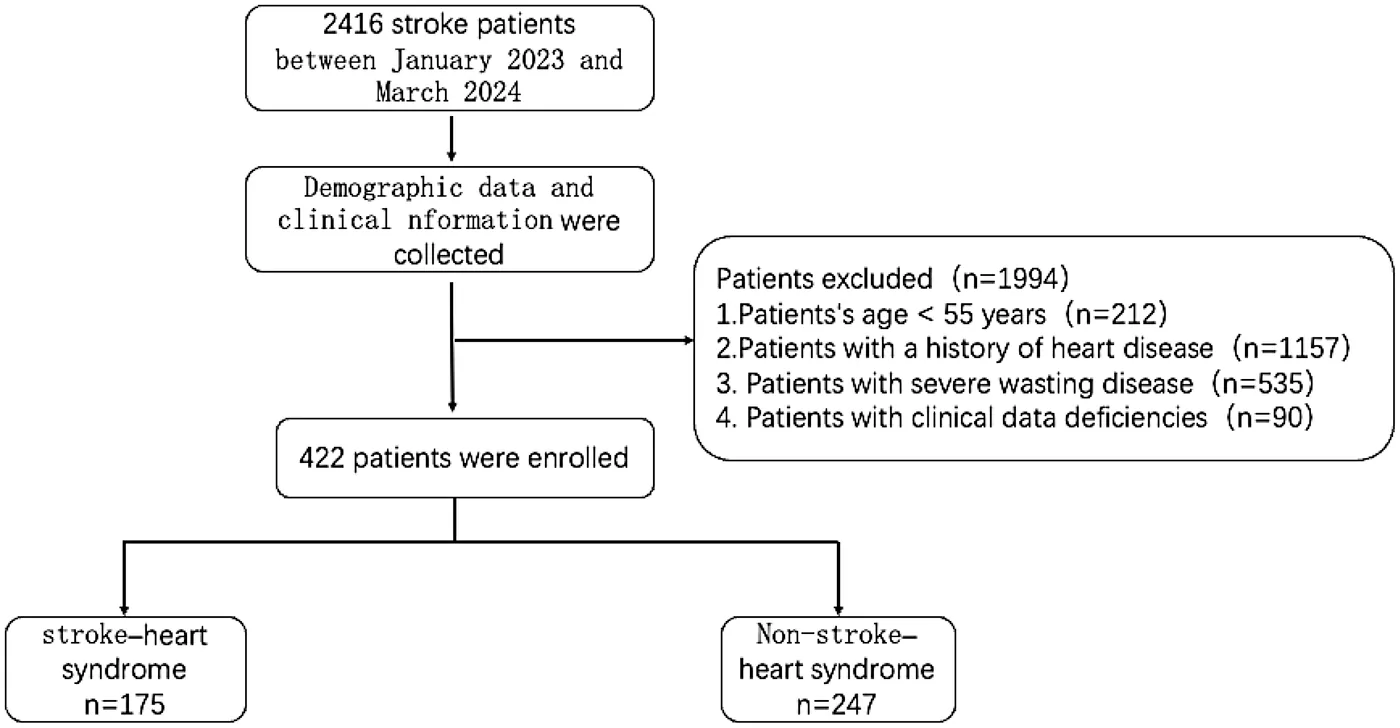
Flow chart of the study population.

Clinical data were retrospectively collected from patients with stroke admitted to Zhejiang Provincial People's Hospital between January 2023 and March 2024. Patients who met the diagnostic criteria for stroke established by the American Stroke Association were included in the study (17). The exclusion criteria were as follows: (1) age <55 years; (2) a history of heart disease; (3) severe wasting conditions, such as malignant tumors, severe infections, or hematological disorders; (4) electrolyte disorders; and (5) incomplete data. Ultimately, 422 patients were included in the final analysis (Figure 1).

### Definitions

SHS is defined as the development of acute myocardial injury, cardiac insufficiency, or arrhythmia within 72 h following acute encephalopathy (4). This syndrome can manifest in five categories: (1) ischemic and non-ischemic acute myocardial injury, with markers of myocardial injury including elevated B-type natriuretic peptide; cardiac troponin TNI, and creatine kinase isoenzymes (CK-MB), which are often asymptomatic; (2) post-stroke acute myocardial infarction; (3) left ventricular dysfunction (ejection fraction <55%, ventricular wall motion abnormalities, etc.), heart failure, and post-stroke Takotsubo syndrome; (4) changes on electrocardiogram (ECG) and arrhythmias, including ventricular arrhythmia, supraventricular tachycardia except sinus tachycardia, sinuatrial block or asystole, second- or third-degree atrioventricular block, and atrial fibrillation (18); and (5) sudden cardiac death of neurogenic origin following stroke (19). Cardiac complications were monitored through continuous tracking of myocardial injury markers, ECG, and echocardiography. Some patients exhibited multiple abnormalities.

### Data collection

Baseline demographic and clinical data were extracted from medical records, including age, sex, height, weight, BMI, heart rate, systolic and diastolic blood pressures (SBP, DBP), smoking and alcohol consumption status, and medication history. Hypertension was defined as SBP ≥ 140 mmHg or DBP ≥ 90 mmHg, based on repeated measurements during hospitalization, or ongoing use of antihypertensive medication. Patients with fasting blood glucose levels ≥7 mmol/L or those who were receiving oral hypoglycemic medication or insulin injections were considered diabetic. Dyslipidemia was characterized by elevated lipid markers, such as triglycerides (TG) or total cholesterol (TC), based on biochemical tests, or the use of lipid-lowering therapy. Stroke history was defined as having experienced a previous stroke.

Laboratory tests conducted on the day of admission included levels of serum albumin; hemoglobin (Hb), C-reactive protein (CRP), TC, TG, low-density lipoprotein cholesterol (LDL-C), high-density lipoprotein cholesterol (HDL-C), alanine transaminase; aspartate aminotransferase (AST), D-dimer, fibrinogen (FIB), serum creatinine (Scr), creatine kinase (CK), CK-MB, B-type natriuretic peptide, and cardiac troponin TNI, as well as the prothrombin time (PT), activated partial thromboplastin time, thrombin time, and estimated glomerular filtration rate (eGFR). Routine ECG and 24-hour ambulatory ECG outcomes were also evaluated.

**Figure 2:**
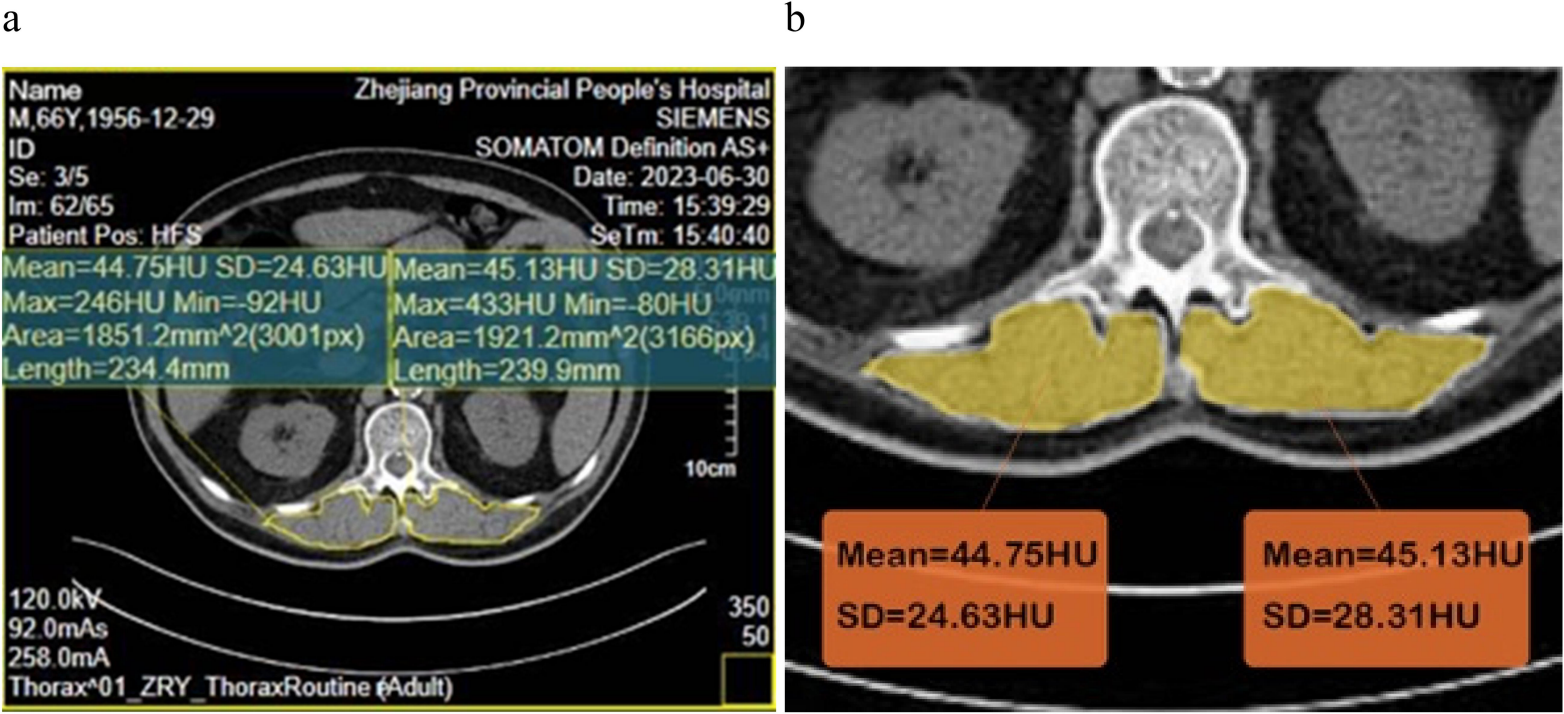
Representative computed tomographic images used to measure the erector spinae muscle density. **(a)** Example of assessing erector spinae muscle density in patients with stroke; **(b)** Drawing the border of the erector spinae muscle density, automatically analyzed by the computer.

CT imaging was employed to assess ESMD, a proxy for the overall muscle mass (20, 21). Cross-sectional scans focused on the T12 or L1 intervertebral discs, and bilateral measurements were averaged to ensure accuracy and reduce bias. CT scans were performed within one month before stroke admission using a 128-slice spiral CT scanner (SIEMENS) with the following parameters: tube voltage of 120 kV, pitch of 1.5, reconstructed layer thickness and spacing of 3 mm, and rotation time of 0.5 s. Imaging analyses were conducted by two independent radiologists blinded to clinical data, who delineated muscle contours manually, with the system calculating average densities, ICC analysis using a two-way random-effects model for absolute agreement. The final ESMD values represented the mean of both radiologists' measurements (Figure 2).

### Statistical analysis

Statistical analyses were conducted using IBM SPSS (version 26.0; IBM Corp, Armonk, NY, USA), and R (version 4.1.0, R Foundation for Statistical Computing, Vienna, Austria) software. Graphs were generated using GraphPad Prism (version 10.0; GraphPad Software, Inc., San Diego, CA, USA). The Kolmogorov–Smirnov test was applied to assess the normality of continuous variables. Normally distributed continuous variables are presented as means ± standard deviation and were analyzed using the Student's *t*-test. Non-normally distributed continuous variables are described as medians (interquartile range) and analyzed using the Mann–Whitney *U*-test. Categorical variables are presented as frequencies (percentages) and were analyzed using the chi-square test.

Variables with *P*-values < 0.1 in baseline comparisons between patients with and without SHS were included in univariate and multivariate logistic regression models. Multicollinearity was evaluated, excluding variables with a tolerance < 0.2 or variance inflation factor >5 from multivariate analysis. ESMD values were stratified into quartiles, and the Mantel–Haenszel chi-square test assessed linear trends. Pearson's correlation coefficient was used to analyze relationships between ESMD and SHS incidence. Trend significance (*P*-values) was determined using logistic regression, with quartile medians treated as quasi-continuous variables. To construct a novel nomogram, R software (version 4.1.0) was used. Receiver operating characteristic (ROC) curve analysis was used to assess the predictive accuracy of the nomogram, while calibration curves were used to evaluate the agreement between actual and predicted probabilities. Decision curve analysis (DCA) was employed to assess the clinical utility of the nomogram. All statistical analyses were two-tailed with 95% confidence intervals (CIs), and *P*-values < .05 were considered statistically significant except for baseline characterization (*P* < 0.1).

## Results

### Baseline characteristics

The baseline characteristics of the study population are summarized in Table 1. A total of 422 patients were included. Patients were categorized into two groups: the non-SHS group (*n* = 247) and the SHS group (*n* = 175) based on the presence of secondary cardiac complications. Compared to the non-SHS group, patients in the SHS group were older, exhibited higher heart rates, and had a significantly higher proportion of hypertension, diabetes mellitus, and stroke history. Additionally, a larger proportion of patients in the SHS group had received prehospital treatment with antiplatelet agents, ACE inhibitors/angiotensin receptor blockers, and calcium channel blockers.

**Table 1:** Patient characteristics.

Variables	Non-Stroke–heart syndrome	Stroke–heart syndrome	P值
	(*n* = 247)	(*n* = 175)	
Demographic characteristics			
Age (years)	68.80 ± 8.52	73.06 ± 9.42	<0.001 *
Male, *n* (%)	164 (66.4%)	109 (62.3%)	0.384 #
Weight (kg)	65.55 ± 10.25 *n* = 174	64.51 ± 11.15 *n* = 100	0.434 *
Height (m)	1.64 ± 0.07 *n* = 174	1.64 ± 0.08 *n* = 100	0.683 *
BMI (kg/m^2^)	24.33 ± 3.46 *n* = 174	24.02 ± 3.50 *n* = 100	0.468 *
Current smoker, *n* (%)	71 (28.7%)	39 (22.3%)	0.136 #
Current drinker, *n* (%)	70 (28.3%)	38 (21.7%)	0.124 #
Vital signs at admission			
Heart rate (bmp)	77.28 ± 12.21	79.57 ± 12.97	0.065 *
SBP (mmHg)	152.20 ± 19.69	154.81 ± 21.86	0.201 *
DBP (mmHg)	83.96 ± 12.49	81.91 ± 13.08	0.104 *
Comorbidities			
Hypertension, *n* (%)	164 (66.4%)	130 (74.3%)	0.082 #
Diabetes, *n* (%)	72 (29.1%)	66 (37.7%)	0.065 #
cerebral stroke, *n* (%)	31 (12.6%)	36 (20.6%)	0.026 #
Medications			
medical treatment, *n* (%)	144 (58.3%)	107 (61.1%)	0.558 #
Anti-plate drugs, *n* (%)	12 (4.9%)	19 (10.9%)	0.02 #
ACEI/ARB, *n* (%)	66 (26.7%)	33 (18.9%)	0.06 #
Beta-blocker, *n* (%)	14 (5.7%)	12 (6.9%)	0.629 #
calcium channel blockers, *n* (%)	73 (29.6%)	67 (38.3%)	0.061 #
diuretic, *n* (%)	20 (8.1%)	10 (5.7%)	0.348 #
lipid-lowering treatment, *n* (%)	13 (5.3%)	13 (7.4%)	0.362 #
hypoglycemic therapy, *n* (%)	58 (23.5%)	53 (30.3%)	0.118 #
Laboratory findings			
CRP (mg/L)	2.00 (1.30–5.00)	3.40 (1.30–9.70)	<0.001 +
Hb (g/L)	137.26 ± 14.60	131.58 ± 17.72	0.001 *
Serum albumin (g/L)	37.67 ± 2.80	36.23 ± 3.47	<0.001 *
Scr (Umol/L)	69.30 (59.70–80.10)	71.90 (60.10–87.90)	0.024 +
eGFR (mL/min × 1.73 m^2^)	89.75 (81.72–96.44)	83.37 (69.13–93.40)	<0.001 +
ALT (U/L)	16.00 (12.00–23.00)	16.00 (12.00–26.00)	0.403 +
AST (U/L)	20.00 (17.00–24.00)	21.00 (17.00–27.00)	0.061 +
CK (U/L)	77.00 (55.00–110.00)	79.00 (53.00–117.75)	0.865 +
D-dimer (*100 *μ*g/L)	3.30 (1.70–6.80)	5.00 (2.60–10.30)	<0.001 +
PT (s)	11.33 ± 0.93	11.75 ± 0.86	<0.001 *
APTT (s)	25.70 (24.50–27.10)	26.20 (24.90–27.60)	0.023 +
TT (s)	16.80 (16.20–17.50)	16.40 (15.73–17.30)	<0.001 +
FIB (g/L)	2.77 ± 0.62	3.55 ± 1.22	<0.001 *
TC (mmol/L)	4.49 ± 1.04	4.44 ± 1.04	0.604 *
TG (mmol/L)	1.25 (0.92–1.72)	1.17 (0.90–1.65)	0.5 +
LDL-C (mmol/L)	2.70 (2.18–3.27)	2.65 (2.11–3.24)	0.885 +
HDL-C (mmol/L)	1.04 (0.88–1.22)	0.98 (0.83–1.14)	0.016 +
Results of lung CT examination			
ESMD (HU)	38.93 ± 13.73	26.41 ± 18.02	<0.001 *

BMI, body mass index; bpm, beats per minute; SBP, systolic blood pressure; DBP, diastolic blood pressure; ACEI, angiotensin-converting enzyme inhibitor; ARB, angiotensin receptor blocker; CRP, C-reactive protein; Hb, hemoglobin; Scr, serum creatinine; eGFR, estimated glomerular filtration rate; ALT, Alanine transaminase; AST, Aspartate aminotransferase; CK, Creatine kinase; PT, Prothrombin time; APTT, activated partial thromboplastin time; TT, thrombin time; FIB, Fibrinogen; TC, total cholesterol; TG, triglyceride; LDL-C, low-density lipoprotein cholesterol; HDL-C, high-density lipoprotein cholesterol; ESMD, erector spinae muscle density.

Laboratory findings revealed that patients in the SHS group exhibited higher levels of CRP, Scr, AST, D-dimer, PT, activated partial thromboplastin time, and FIB (*P* < 0.1), whereas Hb, serum albumin, HDL-C, and ESMD levels, and the eGFR and thrombin time and were lower (*P* < 0.1). No significant differences were observed between the two groups in terms of sex, height, weight, BMI, current smoking or drinking status, SBP, DBP, the proportion of current medication use, β-blocker medication, diuretic use, lipid-lowering and glucose-lowering therapy, and alanine transaminase, CK, TC, TG, and LDL-C levels.

### Clinical predictors of SHS

Variables with a *P*-value < 0.1 were included in the univariate logistic regression analysis, followed by multicollinearity assessment. The results are summarized in Table 2. The risk of developing SHS was significantly associated with a history of previous stroke [odds ratio (OR) = 1.805, 95% CI: 1.067–3.052, *P* = 0.028] and the use of antiplatelet medication (OR = 2.385, 95% CI: 1.126–5.052, *P* = 0.023). In addition, continuous variables, such as age, eGFR, PT, ESMD, and levels of CRP, Hb, serum albumin, Scr, AST, D-dimer, FIB, and HDL-C, were identified as potential clinical predictors. Multicollinearity assessment revealed no strong correlations among these variables.

**Table 2:** Potential clinical predictors for stroke–heart syndrome.

Predictors	Univariate Analysis			Multicollinearity Analysis	
	OR	95% CI	P值	Tolerance	VIF
Age (years)	1.054	1.031–1.078	<0.001	0.727	1.375
Cerebral stroke, *n* (%)	1.805	1.067–3.052	0.028	0.844	1.184
Anti-plate drugs, *n* (%)	2.385	1.126–5.052	0.023	0.83	1.204
CRP (mg/L)	1.028	1.011–1.045	0.001	0.731	1.369
Hb (g/L)	0.978	0.966–0.990	<0.001	0.69	1.449
Serum albumin (g/L)	0.86	0.804–0.919	<0.001	0.744	1.344
Scr (Umol/L)	1.013	1.005–1.022	0.002	0.234	4.271
eGFR (mL/min × 1.73 m^2^)	0.975	0.963–0.986	<0.001	0.222	4.505
AST (U/L)	1.019	1.002–1.035	0.025	0.955	1.047
D-dimer (*100 *μ*g/L)	1.013	1.003–1.024	0.012	0.879	1.047
PT (s)	1.859	1.434–2.410	<0.001	0.865	1.155
FIB (g/L)	3.129	2.321–4.220	<0.001	0.715	1.399
HDL-C (mmol/L)	0.399	0.187–0.851	0.017	0.954	1.048
ESMD (HU)	0.946	0.931–0.961	<0.001	0.713	1.402

CRP, C-reactive protein; Hb, Hemoglobin; Scr, Serum creatinine; eGFR, Estimated glomerular filtration rate; AST, Aspartate aminotransferase; PT, Prothrombin time; FIB, Fibrinogen; HDL-C, High-density lipoprotein cholesterol; ESMD, Erector spinae muscle density; CI, Confidence interval; OR, Odds ratio; VIF, Variance inflation factor; HU, Hounsfield unit.

Subsequently, we incorporated all the identified potential clinical predictors into a multivariate logistic regression model using backward stepwise selection (Backward: LR). The results, shown in Table 3, revealed that after adjusting for confounders, a lower ESMD emerged as an independent and significant risk factor for SHS (OR = 0.946, 95% CI: 0.931–0.961, *P* < 0.001). Furthermore, the multivariate analysis revealed several other significant predictors: serum albumin level (OR = 0.910, 95% CI: 0.833–0.995, *P* = 0.037), eGFR (OR = 0.979, 95% CI: 0.964–0.995, *P* = 0.011), PT (OR = 1.719, 95% CI: 1.214–2.433 (*P* = 0.002), and FIB level (OR = 3.002, 95% CI: 2.106–4.281, *P* < 0.001).

**Table 3:** Independent clinical predictors for stroke–heart syndrome.

Predictors	Multivariate Analysis			
	OR	95% CI	*P*-value	AUC
Serum albumin (g/L)	0.910	0.833–0.995	0.037	0.621
eGFR (mL/min × 1.73 m^2^)	0.979	0.964–0.995	0.011	0.616
PT (s)	1.719	1.214–2.433	0.002	0.636
FIB (g/L)	3.002	2.106–4.281	<0.001	0.728
ESMD (HU)	0.944	0.926–0.963	<0.001	0.741

eGFR, Estimated glomerular filtration rate; PT, Prothrombin time; FIB, Fibrinogen; ESMD, Erector spinae muscle density; CI, Confidence interval; OR, Odds ratio; AUC, Area under the curve; HU, Hounsfield unit.

### ESMD: a good predictor of SHS

**Figure 3:**
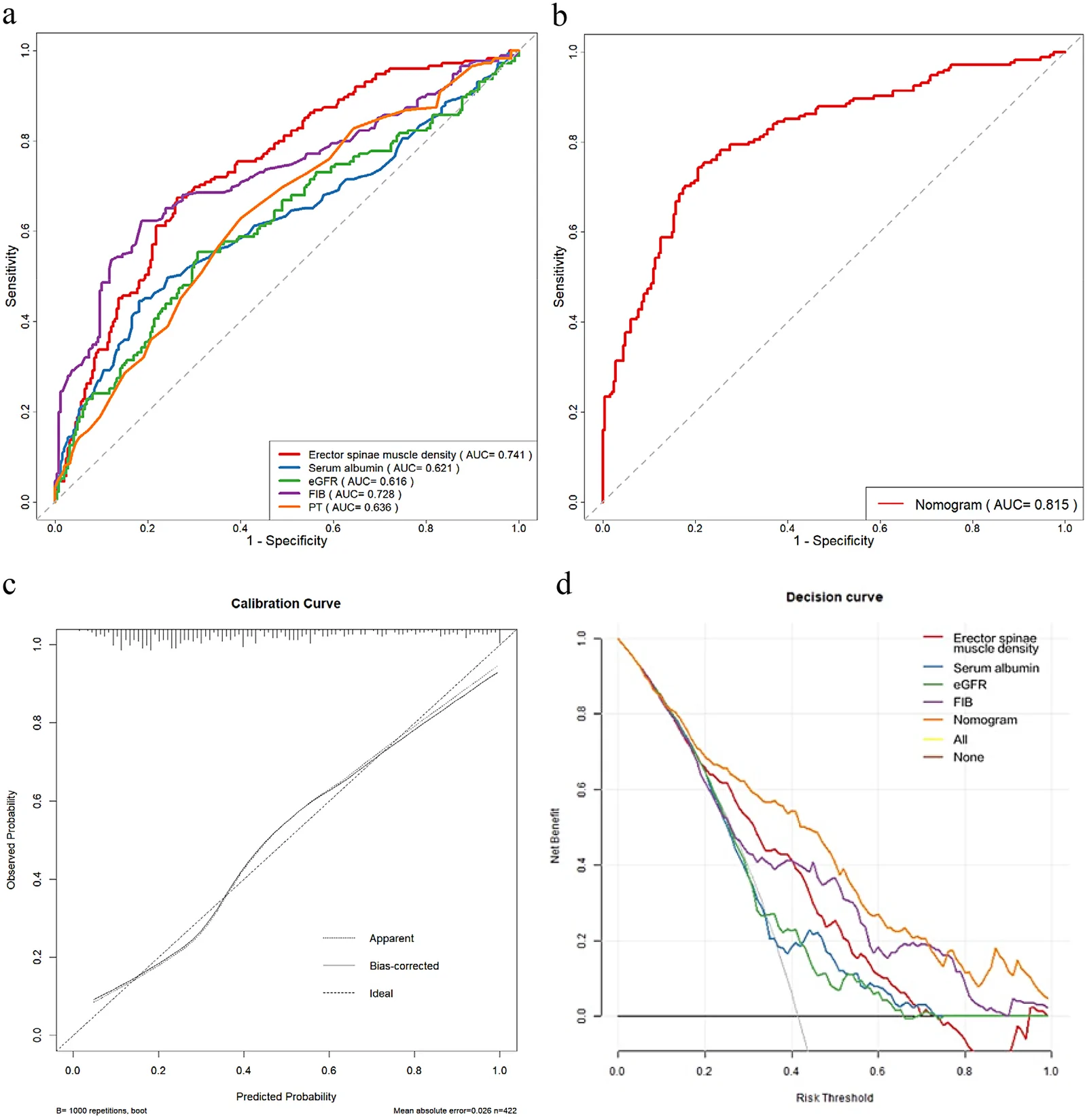
Demonstration of the net benefit of using nomograms. **(a)** Receiver operating characteristic (ROC) curves of erector spinae muscle density; **(b)** ROC curve for the nomogram predicting the incidence of stroke–heart syndrome post-stroke; **(c)** Calibration curve for the nomogram predicting the incidence of stroke–heart syndrome in patients with stroke; **(d)** Decision curve analysis (DCA) for predicting the incidence of new stroke-heart syndrome in patients with stroke.

The ROC curve for ESMD, as shown in Figure 3A, demonstrated its ability to predict the occurrence of SHS, with an area under the curve (AUC) of 0.741 (95% CI: 0.694–0.789). An optimal threshold of <34.39 Hounsfield units (HU) identified patients at risk for SHS, with 67.4% sensitivity and 73.7% specificity, yielding a maximal Youden index (J) of 0.411. The predictive performance of ESMD was superior to that of the serum albumin level (AUC = 0.621, 95% CI: 0.564–0.677), eGFR (AUC = 0.616, 95% CI: 0.560–0.671), PT (AUC = 0.636, 95% CI: 0.582–0.689), and FIB (AUC = 0.728. 95% CI: 0.677–0.779).

To further investigate the relationship, ESMD was stratified into quartiles. The incidence of SHS was 69.4% in patients with an ESMD of <26.41 HU, 48.5% in those with an ESMD of 26.41–36.95 HU, 31.3% in those with an ESMD of 36.95–44.42 HU, and 14.3% in those with an ESMD > 44.42 HU. The Mantel–Haenszel chi-square test revealed a significant linear relationship between ESMD and the incidence of SHS (*P* < 0.001). Additionally, the incidence of SHS showed a negative correlation with ESMD (Pearson's *r* = −0.413, *P* < 0.001). The chi-square test across the four groups showed a statistically significant difference (χ^2^ = 72.318, *P* < 0.001), confirming that the incidence rates were not equal across the quartiles (Table 4). Pairwise comparisons between the groups, adjusted for a significance level of *P* = 0.0083, indicated that the difference between the second and third quartiles (Q2 vs. Q3) was not statistically significant. The analysis showed good inter-rater reliability for ESMD (ICC = 0.82, 95% CI: 0.74–0.88), indicating substantial agreement between the two raters. However, significant differences in the incidence of SHS were observed between all other quartile pairs. These results are visually represented in the Supplementary Figures (Supplementary Figures S1, S2).

**Table 4:** Trend test of erector spinae muscle density level and incidence of stroke–heart syndrome.

Median ESMD (range)	Incidence of stroke–heart syndrome number of samples/total number of patients (%)
Q1: 17.53 (<26.41)	77/111 (69.4%)
Q2: 32.93 (26.41–36.95)	49/101 (48.5%)
Q3: 40.78 (36.95–44.42)	35/112 (31.3%)
Q4: 49.99 (>44.42)	14/98 (14.3%)
P for trend	<0.001

Subgroups of ESMD levels, expressed as the median muscle density (range): Q1: 17.53 (<26.41); Q2: 32.93 (26.41–36.95); Q3: 40.78 (36.95–44.42); Q4: 49.99 (>44.42).

ESMD, erector spinae muscle density.

**Figure 4:**
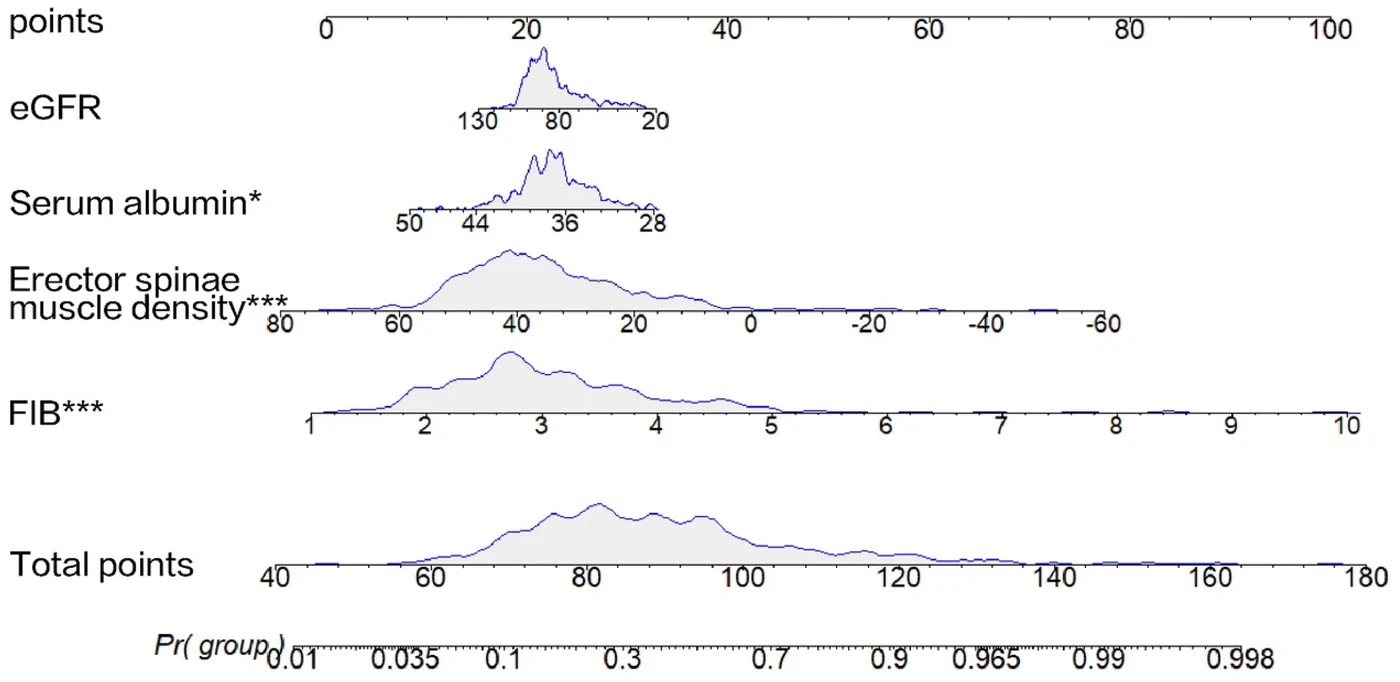
Nomograms for calculating risk scores and predicting the incidence of stroke-heart syndromes in patients with stroke. *P < 0.05, **P < 0.01, ***P < 0.001 indicates significant difference. eGFR, estimated glomerular filtration rate; FIB, fibrinogen.

ESMD, serum albumin level, eGFR, PT, and FIB level were identified as significant predictors of SHS (Table 3). A novel nomogram incorporating these variables was developed (Figure 4). The predictive performance of the nomogram was evaluated using ROC analysis, which yielded an AUC of 0.815 (95% CI: 0.773–0.857), demonstrating excellent discriminatory ability (Figure 3b). Calibration analysis confirmed strong agreement between predicted and observed probabilities, with an average absolute error of 0.026 (Figure 3c). DCA revealed that under the threshold probability of the main cohort, a greater net benefit could be obtained when using the predictive model to make clinical decisions, indicating the clinical practicability of the novel nomogram (Figure 3d).

## Discussion

Our study investigated the potential independent risk predictors of cardiac complications in a cohort of 422 patients hospitalized for acute stroke at Zhejiang Provincial People's Hospital (Hangzhou, China). After adjusting for confounders, we identified ESMD, serum albumin levels, eGFR, PT, and FIB levels as independent predictors of SHS. Among them, ESMD exhibited the highest predictive utility (AUC = 0.741, 95% CI: 0.694–0.789). Notably, higher ESMD values at admission were inversely associated with SHS incidence. Furthermore, a novel nomogram integrating ESMD was developed, achieving robust predictive performance (AUC = 0.815, 95% CI: 0.773–0.857) and validated through calibration curves and DCA. To our knowledge, this represents the first investigation to utilize ESMD as a predictive marker for SHS and to develop an ESMD-based nomogram.

Cardiovascular complications are the second leading cause of post-stroke mortality (5). These complications not only prolong patient hospitalization and increase in-hospital mortality but are also associated with poor long-term prognosis.

Serum albumin levels reflect the nutritional status of an individual (10). A meta-analysis showed a strong independent association between low plasma albumin levels and CVD (22). In addition, albumin levels have been reported to be negatively associated with the risk of early cardiovascular events and death in patients with ischemic stroke (23). On the other hand, high nutritional risk and elevated inflammatory markers are independently associated with hypoalbuminemia (24). Our study corroborates these findings, showing that hypoalbuminemia impairs anti-inflammatory and oxidative stress responses, potentially exacerbating SHS development.

Renal function, measured by the eGFR, is a critical determinant of cardiovascular health. Previous studies, including the Atherosclerosis Risk in Communities study, identified a reduced eGFR as an independent risk factor for atherosclerotic CVD and mortality (25, 26). In our study, a low eGFR was significantly associated with increased SHS risk, even after adjusting for confounders. This relationship may be mediated by oxidative stress and inflammation, which are exacerbated in chronic kidney disease and acute encephalopathy (27).

Similarly, FIB, an essential clotting factor, is considered an independent risk factor for vascular events (28). Landmark studies, such as that by Wilhelmsen et al. and the Framingham study, highlighted its association with CVD (29, 30). This finding is supported by our current study, where patients with stroke exhibited significantly elevated FIB levels as an acute-phase reactant (31). Furthermore, patients with secondary cardiac complications had higher levels of FIB compared to those without cerebral cardiac syndrome. Acute-phase elevations in FIB correlate with higher SHS risk, emphasizing its relevance in stroke-related cardiac complications.

Although PT differences were observed between SHS and non-SHS groups, these values fell within the normal reference range. Antiplatelet therapy was more prevalent in patients with SHS and may have influenced PT values. Due to its limited discriminatory value, PT was not included in our final predictive model.

Nutritional status is a critical determinant of stroke outcomes (9). In the present study, we used paravertebral muscle density as a proxy for assessing the nutritional status of our patients (21). Low muscle mass is an indicator of malnutrition and is one of the diagnostic criteria for this condition (32). Muscle density may be a more informative measure than muscle mass preceding the decline in muscle mass (33). An increase of 1 g/100 mL in lipid concentration results in a decrease of approximately 1 HU in the attenuation value of the muscle membrane (34). Body composition analysis using CT to determine the presence of sarcopenia and myosteatosis has become a popular area of research (21). Adipose tissue secretes pro-inflammatory mediators, such as tumor necrosis factor-α and interleukin-6, which contribute to sarcopenia and systemic inflammation (35). These mechanisms accelerate muscle loss and impair physical function (36, 37). Notably, in the current study, no significant difference was observed in BMI between the patients in the SHS group and those in the non-SHS group (Table 1). However, a significant difference was observed in the ESMD between the two groups; patients with SHS had a higher degree of fatty infiltration, indicative of a chronic inflammatory state (38). Inflammation is an important mechanism in the development of SHS (19). CRP, an indicator of inflammation, was not an independent clinical predictor of cerebrocardiac syndrome in this study, although it did serve as a predictor in univariate analysis (*P* = 0.001). The serum CRP concentration rises above 5 mg/L in approximately 6 h and peaks around 48 h (39). The clinical data collected for this study reflected the patients status at the time of hospital admission. Considering the patients with acute stroke included in this study had an onset time to admission time of <24 h, they may not have reached the time window for CRP elevation. Although CRP was not an independent predictor of SHS, its role in the inflammatory cascade warrants further investigation. Temporal dynamics of CRP elevation in patients with stroke should be explored in future studies. Our findings showed that lower ESMD values were associated with higher SHS incidence, likely due to systemic inflammation and reduced muscle quality. Therefore, ESMD provides a practical and precise measure for assessing nutritional and inflammatory states in patients with stroke.

In a prospective cohort study, the Multi-Ethnic Study of Atherosclerosis, muscle density was found to be negatively associated with the risk of cardiovascular events and all-cause mortality (9). Cross-sectional studies using large datasets have also shown a strong correlation between low muscle density and poor clinical outcomes, with low muscle mass increasing the risk of all-cause and CVD mortality (40). Our study's findings add to this evidence by demonstrating that patients with stroke who have a low ESMD are more likely to experience cardiac complications.

ESMD, assessed through CT, offers a reliable and sensitive measure of nutritional status and inflammation. Our stratified analysis revealed a dose-dependent protective effect of higher ESMD against SHS, highlighting its utility in clinical risk stratification. Unlike traditional metrics, ESMD provides a non-invasive, practical tool for early identification of high-risk patients, enabling timely intervention and improved management of stroke-related cardiac complications (41). Nomograms are widely used to predict individual probabilities of clinical events by integrating multiple variables (42). Our nomogram exhibited high clinical predictive performance and value as a practical tool. Furthermore, DCA revealed greater net benefits and high clinical efficiency when using the predictive model for clinical decision-making. The nomogram could identify patients at high risk of SHS and facilitate early intervention to improve clinical outcomes.

However, this study has certain limitations. First, the sample size was relatively small, which limits the generalizability of our findings to the broader population of patients with stroke. Notably, NIHSS were not available for inclusion as covariates in our analyses. This is an important omission, as both factors may act as potential confounders in the relationship between ESMD and SHS.patients with a history of cardiac disease were excluded, and further studies are needed to assess the impact of ESMD on SHS in individuals with pre-existing heart conditions. Additionally, this study did not collect long-term follow-up data, preventing an analysis of how changes in ESMD affect long-term prognosis and adverse outcomes in patients with stroke. Third, We now state that although some degree of subjectivity is inherent, the good inter-rater reliability observed (ICC = 0.82) indicates that the measurements are sufficiently reproducible, which mitigates this concern. Finally, as a single-center retrospective study, the association between ESMD and SHS warrants further validation in large, multicenter studies.

In conclusion, our study is the first to demonstrate that ESMD serves as an independent predictor of secondary SHS in patients with stroke. Furthermore, muscle density is negatively and linearly correlated with the incidence of SHS. Additionally, a novel columnar line graph that incorporates ESMD showed strong predictive performance for secondary SHS following stroke.

## Data Availability

The original contributions presented in the study are included in the article/Supplementary Material, further inquiries can be directed to the corresponding author/s.
