# Supplementary material for: Erector spinae density as a predictor of early cardiac complications in patients with stroke
Source: Front Cardiovasc Med. 2026 Aug 12;13:1878270. doi: 10.3389/fcvm.2026.1878270 (PMC13507670; doi:10.3389/fcvm.2026.1878270)
Supplement: Supplementary file 1 [file Datasheet1.pdf]

# 涉及人的生命科学和医学研究伦理审查批件

## Ethics Committee Approval Letter of Biomedical Research Involving Humans

批件号 **Approval NO.**：浙人医伦审 **2024** 其他第（**239**）号

| | | | |
|---|---|---|---|
| 研究名称<br>**Study Title** | 脑卒中患者继发脑心综合征的一个新的预测指标：竖脊肌密度 | | |
| 申办方<br>**Sponsor** | 浙江省人民医院 | | |
| 受理号<br>**Acceptance Number** | QT2024236 | | |
| 主要研究者<br>**Principal Investigator** | 王利宏 | 承担科室<br>**Responsible Department** | 心血管内科 |
| 审查类别<br>**Category of Review** | 初始审查 | 审查方式<br>**Type of Review** | 快速审查 |
| 审查日期<br>**Date of Review** | 2024 年 10 月 14 日 | 审查地点<br>**Location of Review** | 无 |
| 审查文件清单<br>**Items Reviewed** | 1. 临床科研项目伦理审查申请表<br>2. 研究方案（V1.0/2024-09-27）<br>3. 免除/免签知情同意书申请表<br>4. 主要研究者简历 | | |
| 审评意见<br>**Evaluation** | 无 | | |
| 审查决定<br>**Decision** | 委员会对该研究的审查决定为： ☒同意 (Approval) | | |
| 主任/副主任委员签字<br>**Chair Signature** | | | |
| 签发日期<br>**Date of issue** | 2024.10.15 | | |
| 伦理委员会<br>**Stamp of EC** | 伦理委员会盖章 | | |
| 批件有效期<br>**Period of Validity** | 自本伦理委员会初始审查批准之日起一年内，本临床研究应在本院启动。逾期未启动的，本批件自行废止。 | | |
| 年度/定期跟踪审查<br>**Continue Review** | 审查频率为该研究批准之日起每 12 月一次，首次请于 2025 年 10 月 13 日前 1 个月递交研究进展报告。<br>伦理委员会有根据实际进展情况改变跟踪审查频率的权利。 | | |
| 声明<br>**Statement** | 本伦理委员会的职责、人员组成、操作程序及记录遵循《涉及人的生命科学和医学研究伦理审查办法》、《涉及人的健康相关研究国际伦理准则》、《赫尔辛基宣言》、GCP 和 ICH-GCP 等国际伦理指南和国内相关法律法规。 | | |

注意事项：

1. 请遵循我国相关法律、法规和规章中的伦理原则。
2. 请遵循经本伦理委员会批准的临床研究方案、知情同意书、招募材料等开展本研究，保护研究参与者的健康与权利。对研究方案、知情同意书和招募材料等的任何修改，均须得到本伦理委员会审查同意后方可实施。
3. 在本院发生的SAE/SUSAR以及研发期间安全性更新报告须按照NMPA/GCP最新要求及时递交本伦理委员会，国内外其它中心发生的SAE/SUSAR需定期汇总、评估后递交本伦理委员会。
4. 根据报告情况，本伦理委员会有权对其评估做出新的决定。
5. 自今日起，无论研究开始与否，请在跟踪审查日到期前1个月提交研究进展报告。
6. 申办方应当向牵头机构伦理委员会提交中心研究进展报告汇总；当出现任何可能显著影响研究进行或增加研究参与者危险的情况时，请申请人及时向本伦理委员会提交书面报告。
7. 研究纳入了不符合纳入标准或符合排除标准的研究参与者，符合中止研究规定而未让研究参与者退出研究，给予错误治疗或剂量，给予方案禁止的合并用药等没有遵从方案开展研究的情况；或可能对研究参与者的权益或健康以及研究的科学性造成不良影响等违背GCP原则的情况，请申办方、监查员或研究者提交违背方案报告。
8. 申请人暂停或提前终止临床研究，请及时提交暂停或终止研究报告。
9. 完成临床研究，请申请人提交结题报告。
10. 凡涉及中国人类遗传资源采集标本、收集数据等研究，必须获得中国人类遗传资源管理办公室批准后方可在本中心开展研究。
11. 凡经本伦理委员会批准的研究在实施前，申请人应按相关规定在国家卫健委、药审中心等的临床研究登记备案信息系统平台登记研究相关信息。

1984 ZHEJIANG PROVINCIAL PEOPLE'S HOSPITAL
